# Niche specificity and functional diversity of the bacterial communities associated with *Ginkgo biloba* and *Panax quinquefolius*

**DOI:** 10.1038/s41598-021-90309-0

**Published:** 2021-05-24

**Authors:** Hanan R. Shehata, Subramanyam Ragupathy, Thomas A. Henry, Steven G. Newmaster

**Affiliations:** 1 NHP Research Alliance, College of Biological Science , University of Guelph, Guelph, ON Canada; 2 Department of Microbiology, School of Pharmacy, Mansoura University, Mansoura, Egypt

**Keywords:** Ecology, Microbiology, Plant sciences

## Abstract

Plant-associated bacteria can establish mutualistic relationships with plants to support plant health. Plant tissues represent heterogeneous niches with distinct characteristics and may thus host distinct microbial populations. The objectives of this study are to investigate the bacterial communities associated with two medicinally and commercially important plant species; *Ginkgo biloba* and *Panax quinquefolius* using high Throughput Sequencing (HTS) of 16S rRNA gene*,* and to evaluate the extent of heterogeneity in bacterial communities associated with different plant niches. Alpha diversity showed that number of operational taxonomic units (OTUs) varied significantly by tissue type. Beta diversity revealed that the composition of bacterial communities varied between tissue types. In *Ginkgo biloba* and *Panax quinquefolius,* 13% and 49% of OTUs, respectively, were ubiquitous in leaf, stem and root. Proteobacteria, Bacteroidetes, Actinobacteria and Acidobacteria were the most abundant phyla in *Ginkgo biloba* while Proteobacteria, Bacteroidetes, Actinobacteria, Plantomycetes and Acidobacteria were the most abundant phyla in *Panax quinquefolius*. Functional prediction of these bacterial communities using MicrobiomeAnalyst revealed 5843 and 6251 KEGG orthologs in *Ginkgo biloba* and *Panax quinquefolius*, respectively. A number of these KEGG pathways were predicted at significantly different levels between tissues. These findings demonstrate the heterogeneity, niche specificity and functional diversity of plant-associated bacteria.

## Introduction

Plants live in association with microbial communities, consisting of bacteria, archaea and fungi^1^. Of special interest are plant-associated bacteria, which live inside plants (endophytes), on plant surfaces (epiphytes), or in association with plant roots (rhizosphere bacteria)^1^. Plant associated bacteria may establish a mutualistic relationship with a plant^1^ such that the plant provides nutrients and space for its associated bacteria while associated bacteria support plant health by helping the plant acquire nutrients and resist pathogens. Plant associated bacteria produce important bioactive secondary metabolites including antimicrobials and antioxidants^2,3^, and can also affect secondary metabolite production by the plant^1,2^. A well-known example of such a symbiotic relationship is nitrogen-fixing *Rhizobium* in leguminous plants^4^.

Plants exhibit spatial variation in many characteristics. For example, leaves differ from stems or roots in structure, anatomy, access to nutrients and environmental conditions^5^. The different plant parts represent heterogeneous niches for associated bacteria. Each niche has its distinct characteristics and may thus host its distinct microbial population^5^. This heterogeneity may drive the tissue specificity of some plant associated bacterial communities^6^. Localization in specific plant tissues may also be required for plant-associated bacteria to function optimally^1^.

The diversity and distribution of plant associated bacteria in the different plant tissues are not well understood with only few studies that investigated the spatial variation of bacterial communities within plants^5,7^. Studying the spatial variation of plant associated bacteria may provide clues to the functional roles of the different bacterial taxa in the symbiotic relationship with plants^5^. It can also help understand the role that plant characteristics and environmental factors play in shaping this spatial variation of associated bacteria.

The objective of this study is to investigate the bacterial communities associated with two important medicinal plant species; *Ginkgo biloba* and *Panax quinquefolius. Ginkgo biloba* and *Panax quinquefolius* are two medicinally and commercially important species, which are commonly used in Natural Health Products (NHP). The investigation includes characterization of the profiles of bacterial communities associated with the different plant tissues (leaf, stem, root)*,* to evaluate the extent of heterogeneity in bacterial communities between the different plant niches*,* and to predict potential functional pathways in these bacterial communities.

*Ginkgo biloba* is a very important medicinal plant, that is considered as the most ancient tree on Earth^8^. This species and its active ingredients were described in the literature for their diverse therapeutic effects including cardioprotective^9^, anxiolytic^10^, neuroprotective^11^, cognition and memory enhancer^12–14^, anticancer^15^, anti-metastatic^16^, and anti-inflammatory activities^17^. The main active components in *G. biloba* are terpene trilactones (ginkgolides A, B, C, J, M and bilobalide) and flavonoids (flavones, flavonols, tannins, biflavones, and glycosides of quercitin and kaempferol)^18^. *G. biloba* leaf extracts are widely used in Europe as herbal remedies and in the USA as dietary supplements^19^. Indeed, *G. biloba* EGb 761, which is a dried green leaf extract, is one of the top-selling herbal medicine worldwide^20^, used for the treatment of cardiovascular and neurological disorders^21^.

*Panax quinquefolius* (American ginseng) is another popular NHP with differing pharmacologically active tissues^22^. Ginseng root was reported to have cardioprotective^23^, antihypertensive^23^, immunomodulatory^24,25^, neuroprotective, central nervous system modulatory^25,26^, antitumor^25,26^, and anti-diabetic^25,26^ effects. The activity is mainly attributed to its content of triterpene glycosides, including the protopanaxadiol and the protopanaxatriol ginsenosides^22,26^.

In this study, High Throughput Sequencing (HTS) was used to characterize *Ginkgo biloba* and *Panax quinquefolius* associated microbiota^27–29^. As the vast majority of microbes are not easy to culture using common laboratory conditions^27,30^, HTS is commonly used to characterize plant associated microbiota. HTS generates millions of sequencing reads in a single run and hence facilitates the investigation of complex microbial communities. The HTS sequence data was analyzed to determine alpha diversity (taxonomic richness, Chao-1, Simpson’s index and Shannon’s index), beta diversity (Principle coordinates analysis, Bray–Curtis) and taxonomic abundance of bacterial taxa in *Ginkgo biloba* (GB) and *Panax quinquefolius* (PQ) tissues. Functional prediction of the bacterial communities based on 16S rRNA HTS data was also investigated.

## Results

Considerable microbial diversity was recorded from the sequencing results. Sequencing of 24 samples resulted in 3279574 reads. Quality trimming and adapter trimming resulted in 3274322 reads. Fixed length trimming resulted in 3172566 reads trimmed to 251 bases. Following removal of chimeric reads, 1382861 reads from GB samples were assigned to 4100 OTUs and 1240075 reads from PQ samples were assigned to 4195 OTUs. Removal of OTUs with low abundance (combined abundance less than 50) yielded 923 and 1057 OTUs for GB and PQ, respectively. After removal of OTUs belonging to host DNA, 916 and 1040 OTUs from GB and PQ, respectively, were used in further analyses.

### Alpha diversity of the bacterial communities in the different tissue types

**Figure 1:**
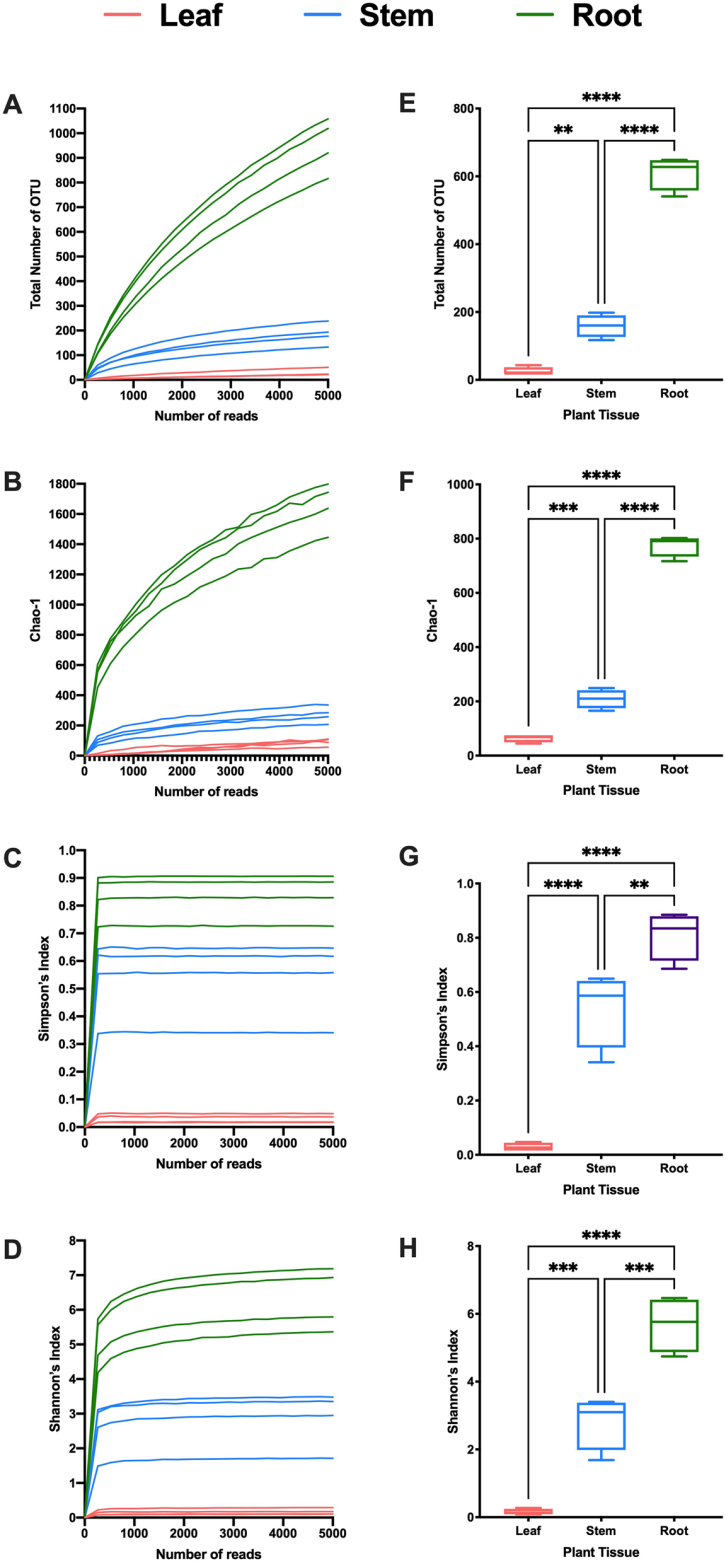
Alpha diversity in *Ginkgo biloba* tissues. (**A**–**D**) Rarefaction curves indicating sufficient depth of sequencing based on (**A**) Total number of operational taxonomic units (OUT), (**B**) Chao-1 index, (**C**) Simpson’s index, and (**D**) Shannon’s index. (**E**–**H**) Alpha diversity in *Ginkgo biloba* tissues based on (**E**) Total number of OTU, (**F**) Chao-1 index, (**G**) Simpson’s index, and (**H**) Shannon’s index.

**Figure 2:**
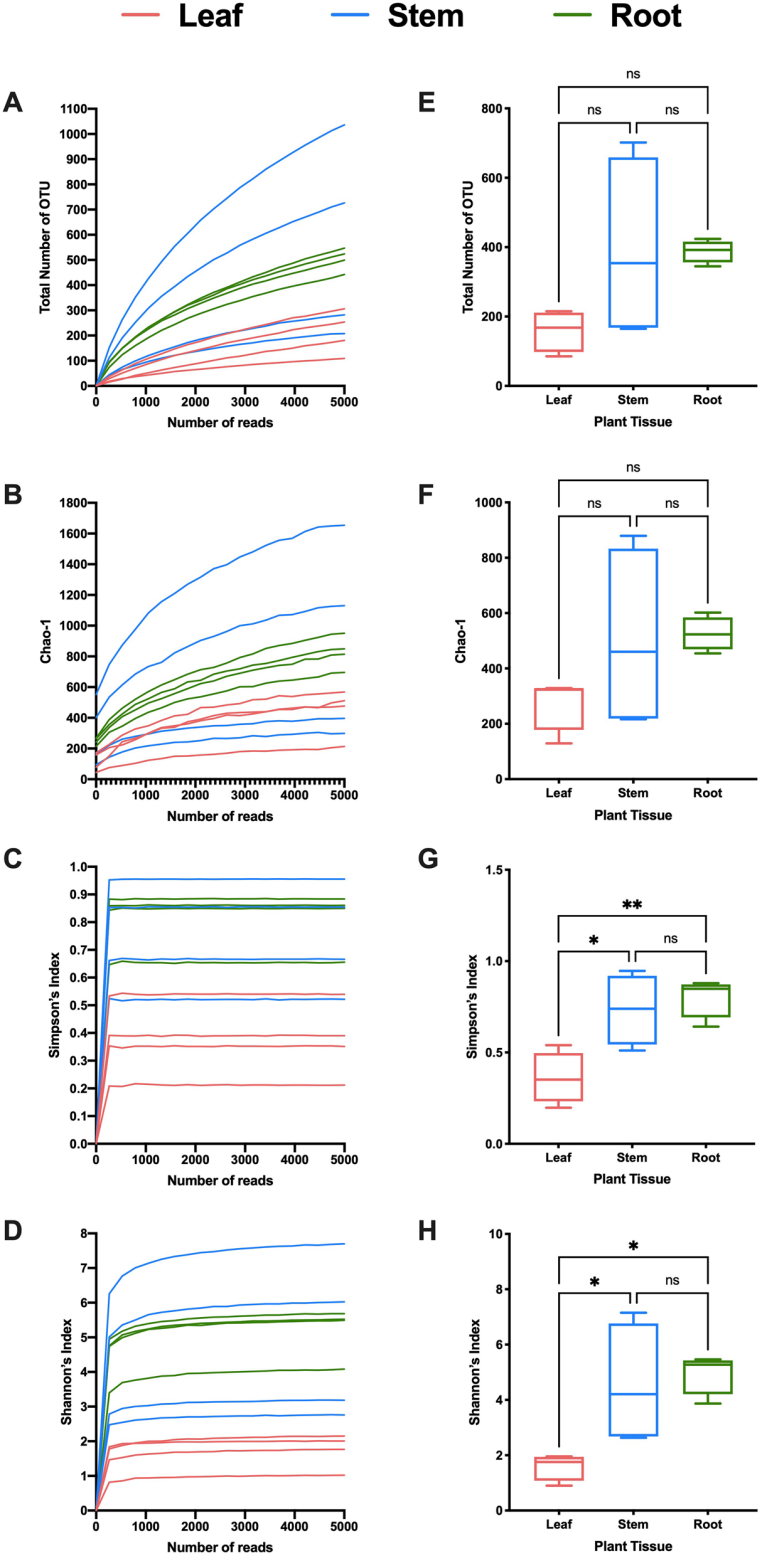
Alpha diversity in *Panax quinquefolius* tissues. (**A**–**D**) Rarefaction curves indicating sufficient depth of sequencing based on (**A**) Total number of operational taxonomic units (OUT), (**B**) Chao-1 index, (**C**) Simpson’s index, and (**D**) Shannon’s index. (**E**–**H**) Alpha diversity in *Panax quinquefolius* tissues based on (**E**) Total number of OTU, (**F**) Chao-1 index, (**G**) Simpson’s index, and (**H**) Shannon’s index.

Sampling of alpha diversity revealed considerable diversity among the tissue types within and among species. Alpha diversity was estimated using total number of OTUs, Chao-1, Simpson’s index, and Shannon’s index. Total number of OTUs and Chao-1 index showed that a deeper sequencing depth may be needed to fully capture the diversity of plant-associated microbiota in GB (Fig. 1A,B) and PQ (Fig. 2A,B). However, Simpson’s index and Shannon’s index showed sufficient sequence coverage using sampling (sequence read) intensity curves in GB (Fig. 1C,D) and PQ (Fig. 2C,D).

In GB, the total number of OTUs varied significantly by tissue type (ANOVA P value < 0.0001). Multiple comparisons showed that total number of OTUs in root was significantly higher than that in leaf (P value < 0.0001) and stem (P value < 0.0001) (Fig. 1E). Total number of OTUs in stem was significantly higher than that in leaf (P value = 0.0012). Analysis of other alpha diversity metrics (Chao-1, Shannon, Simpson’s index) yielded similar results (Fig. 1F–H).

The ginseng (PQ) samples showed a different pattern where the total number of OTU was not significantly different between the different tissue types (ANOVA P value = 0.1096) (Fig. 2E). Similarly, Chao-1 index showed no significant difference (ANOVA P value = 0.2185 (Fig. 2F). However, Simpson’s index and Shannon index showed significant difference (ANOVA P value = 0.0055 and 0.0135, respectively) (Fig. 2G,H). According to Simpson’s index, PQ leaf has significantly lower diversity compared to stem (P value = 0.0176) and root (P value = 0.0066) (Fig. 2G). Shannon index showed similar results where PQ leaf has significantly lower diversity compared to stem (P value = 0.0334) and root (P value = 0.0171) (Fig. 2H).

### Beta diversity of the bacterial communities in the different tissue types

**Figure 3:**
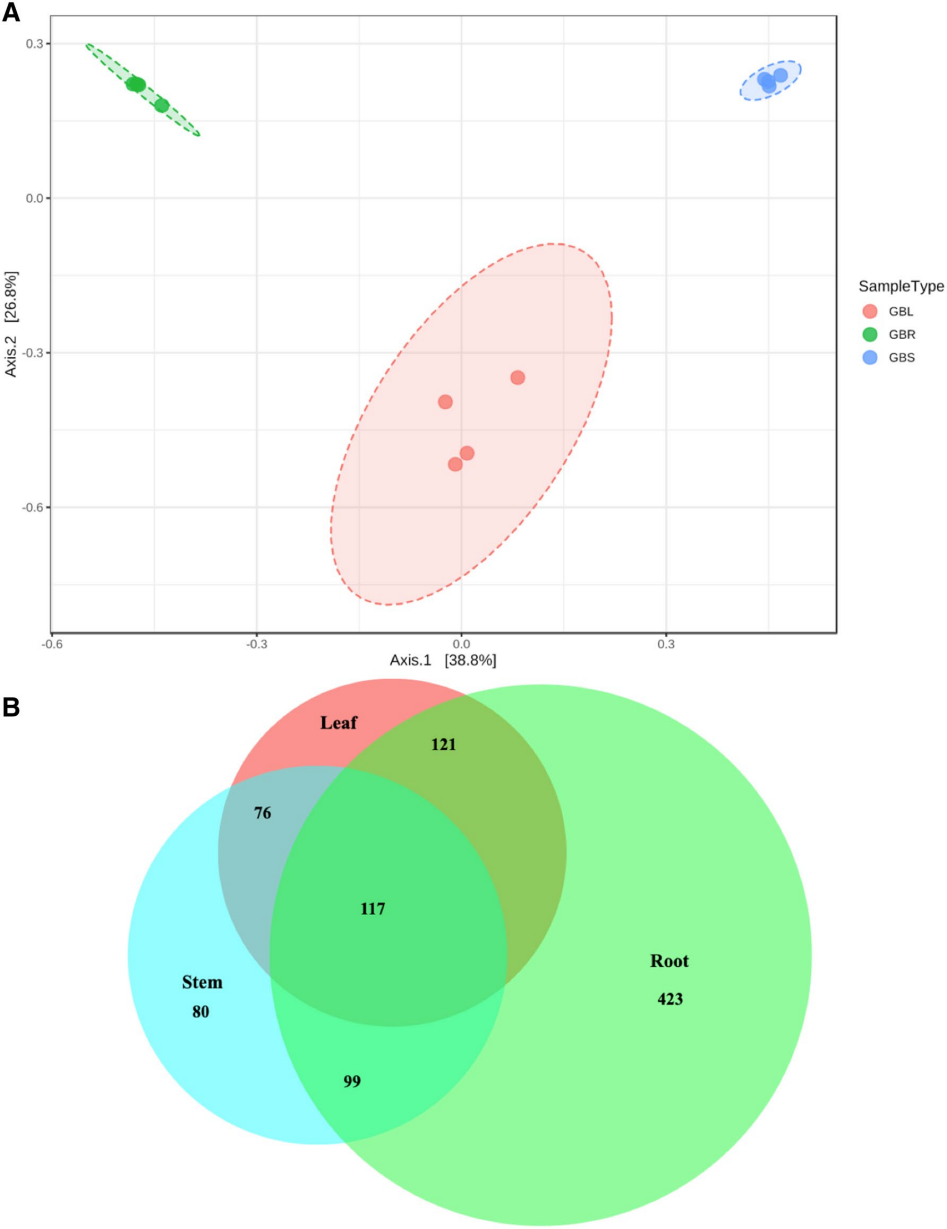
Beta diversity in *Ginkgo biloba* tissues (**A**) Principle coordinates analysis (PCoA) based on Bray–Curtis Index (**B**) Venn diagram demonstrating the proportions of unique and overlapping operational taxonomic units (OTUs) between the different tissues. Venn diagrams were created using BioVenn online tool (http://www.biovenn.nl). GBL, GBR, and GBS refer to *Ginkgo biloba* leaf, root and stem, respectively.

**Figure 4:**
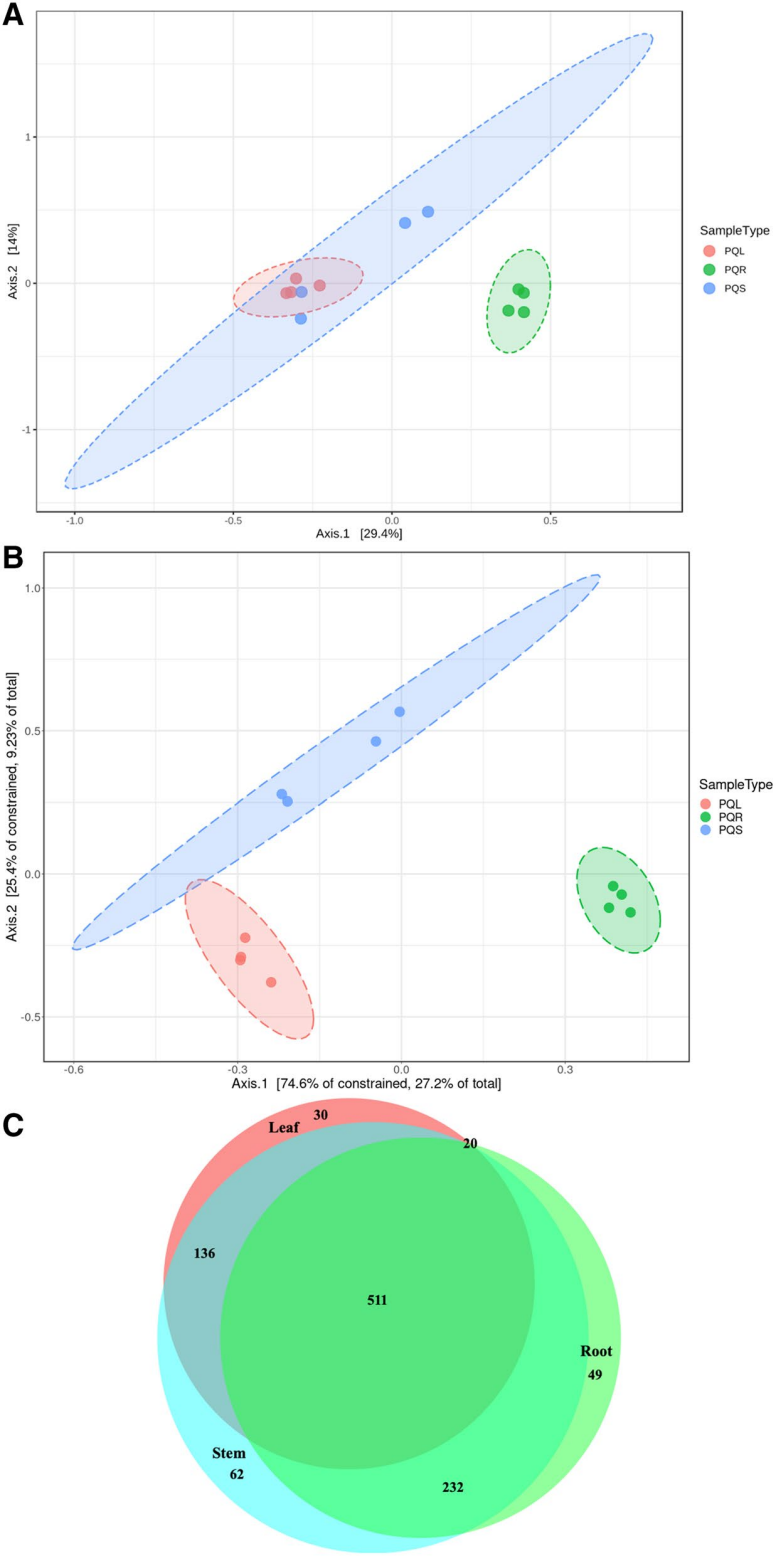
Beta diversity in *Panax quinquefolius* tissues (**A**) Principle coordinates analysis (PCoA) based on Bray–Curtis Index (**B**) Distance-based redundancy analysis (dbRDA) based on Bray–Curtis Index and constrained by tissue type (**C**) Venn diagram demonstrating the proportions of unique and overlapping operational taxonomic units (OTUs) between the different tissues. Venn diagrams were created using BioVenn online tool (http://www.biovenn.nl). PQL, PQR, and PQS refer to *Panax quinquefolius* leaf, root and stem, respectively.

There was considerable variation in community composition among the tissue types for both species. The composition of bacterial communities varied between GB tissue types (Bray–Curtis Permanova P-value < 0.0001) (Fig. 3A). Similarly, bacterial communities varied between PQ tissue types (Bray–Curtis Permanova P-value < 0.0001) (Fig. 4A). For PQ, distance-based redundancy analysis (dbRDA) showed that 36.4% of variation in community composition could be explained by tissue type (Fig. 4B).

The extent to which OTUs were unique or ubiquitous among GB different tissues was investigated. Out of 916 OTUs, 117 OTUs (13%) were ubiquitous in leaf, stem and root (Fig. 3B), 80 OTUs were unique to stem, 423 OTUs were unique to root, 99 OTUs overlapped between root and stem, 121 OTUs overlapped between root and leaf, and 76 OTUs overlapped between leaf and stem (Fig. 3B). The extent to which OTUs were unique or ubiquitous among PQ different tissues was also investigated. Out of 1040 OTUs, 511 OTUs (49%) were ubiquitous in leaf, stem and root (Fig. 4C), 62 OTUs were unique to stem, 30 OTUs were unique to leaf, 49 OTUs were unique to root, 232 OTUs overlapped between root and stem, 20 OTUs overlapped between root and leaf, and 136 OTUs overlapped between leaf and stem (Fig. 4C).

### Microbiota profiles in GB and PQ tissues and variability in taxa diversity among the different tissue types

GB tissues were found to host 916 bacterial taxa belonging to 227 species in 211 genera in 151 families in 18 phyla. PQ tissues were found to host 1040 bacterial taxa belonging to 291 species in 264 genera in 163 families in 19 phyla. Microbiome taxonomic diversity varied among plant tissues in which there were tissue-specific patterns in community assemblage.

**Figure 5:**
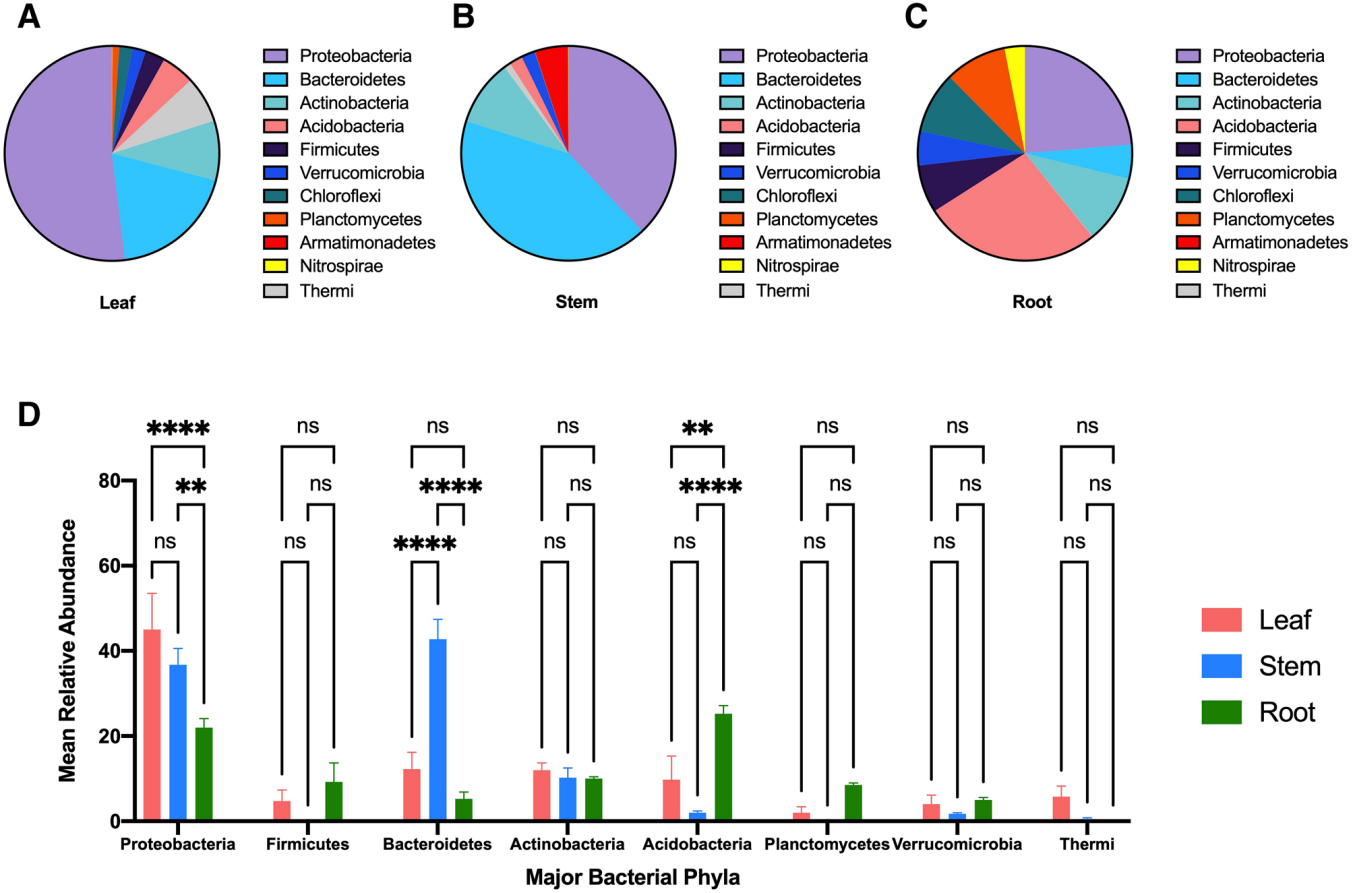
The most abundant bacterial phyla in *Ginkgo biloba* tissues (**A**) Leaf, (**B**) Stem, (**C**) Root. (**D**) The mean relative abundance of the most abundant phyla in *Ginkgo biloba* tissues*.* The mean relative abundance is shown as columns with Standard Error of Mean (SEM).

At the bacterial phyla level, Proteobacteria was the most abundant phylum in GB leaf samples at 52% relative abundance, followed by Bacteroidetes at 19% and Actinobacteria at 9% relative abundance (Fig. 5A). The pattern was different in GB stem where the most abundant phyla were Bacteroidetes at 42% relative abundance, followed by Proteobacteria at 38%, and Actinobacteria at 10% relative abundance (Fig. 5B). The major phyla in GB root were Acidobacteria at 26% relative abundance, Proteobacteria at 23%, and Actinobacteria at 10% relative abundance (Fig. 5C). Three phyla showed significant differences in their relative abundance among GB tissues (Fig. 5D). GB stem had significantly higher relative abundance of Bacteroidetes compared to leaf and root (ANOVA P-value < 0.0001) (Fig. 5D). GB root had significantly higher relative abundance of Acidobacteria compared to leaf and stem (ANOVA P-value = 0.0013 and < 0.0001) and significantly lower relative abundance of Proteobacteria compared to stem and leaf (ANOVA P-value = 0.0024 and < 0.0001) (Fig. 5D).

**Figure 6:**
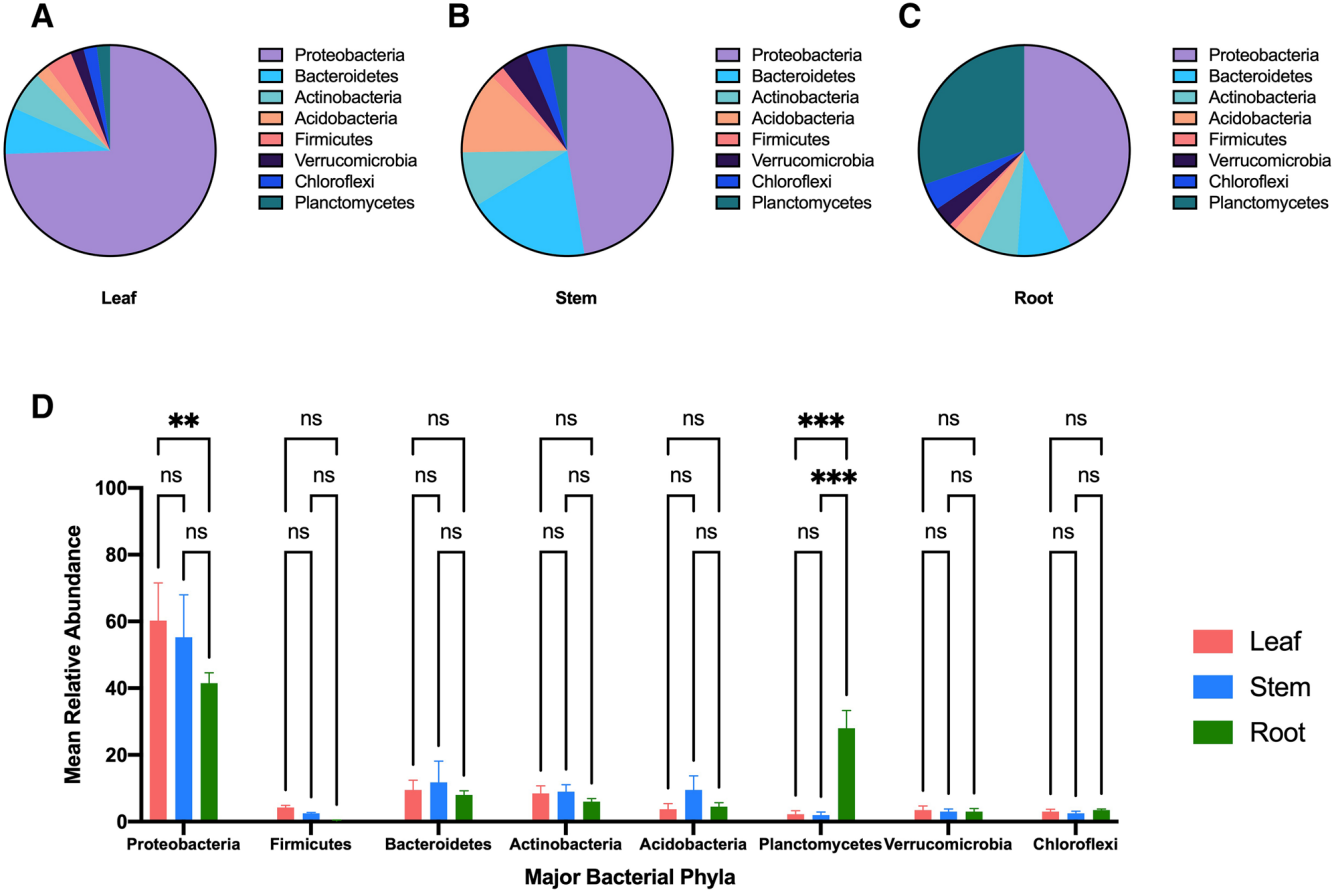
The top bacterial phyla in *Panax quinquefolius* tissues (**A**) Leaf, (**B**) Stem, (**C**) Root. (**D**) The mean relative abundance of the most abundant phyla in *Panax quinquefolius* tissues. The mean relative abundance is shown as columns with Standard Error of Mean (SEM).

The microbiome in ginseng (PQ) had a different assemblage of taxa than what was found in ginkgo. Proteobacteria was the most abundant phylum in all PQ tissues with relative abundance of 73%, 45%, and 41% in PQ leaf, steam and root, respectively (Fig. 6A–C). Other abundant phyla in PQ leaf were Bacteroidetes at 7% and Actinobacteria at 6% relative abundance (Fig. 6A), and in PQ stem were Bacteroidetes at 18%, Acidobacteria at 12% and Actinobacteria at 8% relative abundance (Fig. 6B), and in root were Plantomycetes at 29%, Bacteroidetes at 8%, and Actinobacteria at 6% relative abundance (Fig. 6C). Two phyla showed significant differences in relative abundance among PQ tissues (Fig. 6D). The relative abundance of Plantomycetes was significantly higher in root compared to leaf and stem (ANOVA P-value = 0.0001) (Fig. 6D). The relative abundance of Proteobacteria was significantly higher in leaf compared to root (ANOVA P-value = 0.0060) (Fig. 6D).

**Figure 7:**
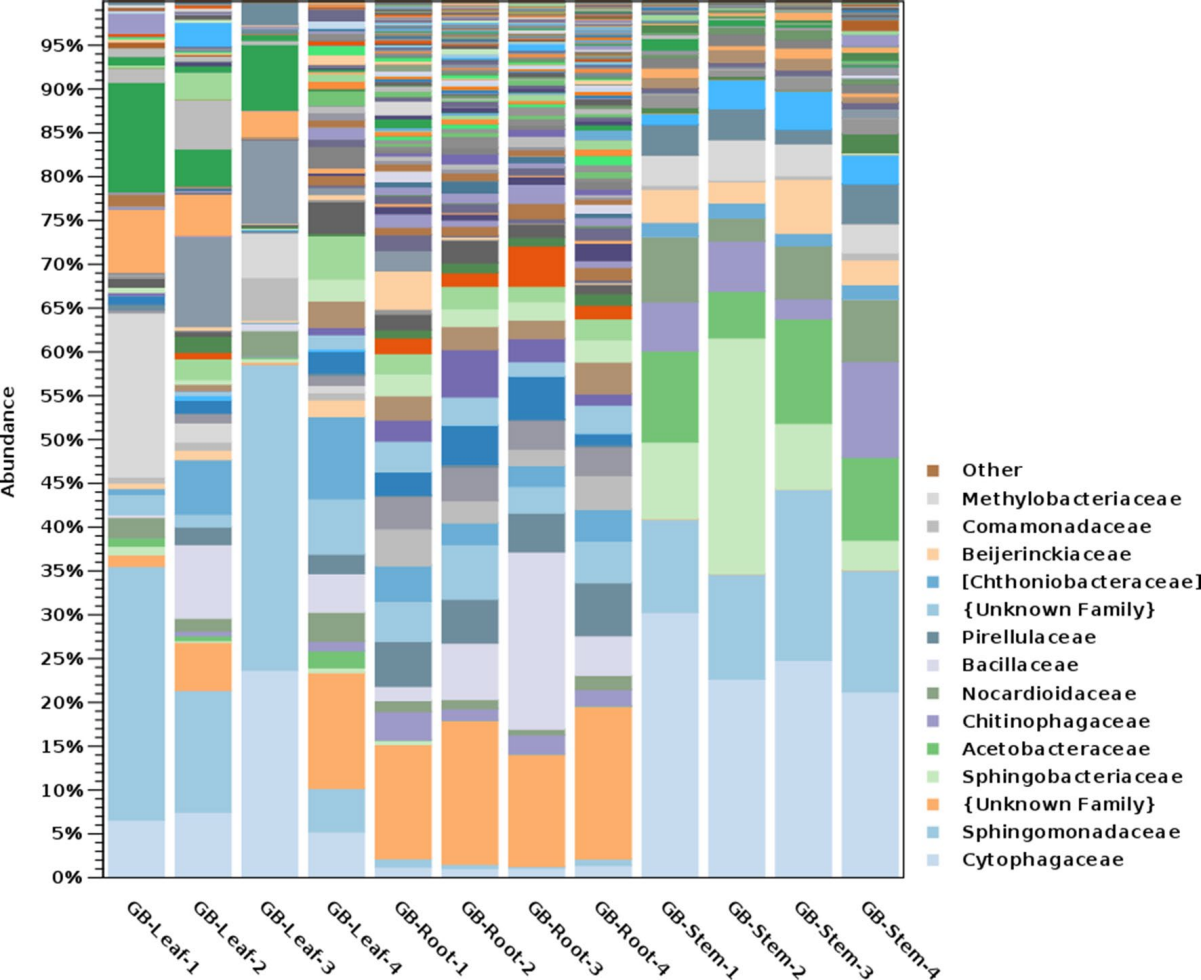
Taxonomic abundance of bacterial families in *Ginkgo biloba* tissues.

**Figure 8:**
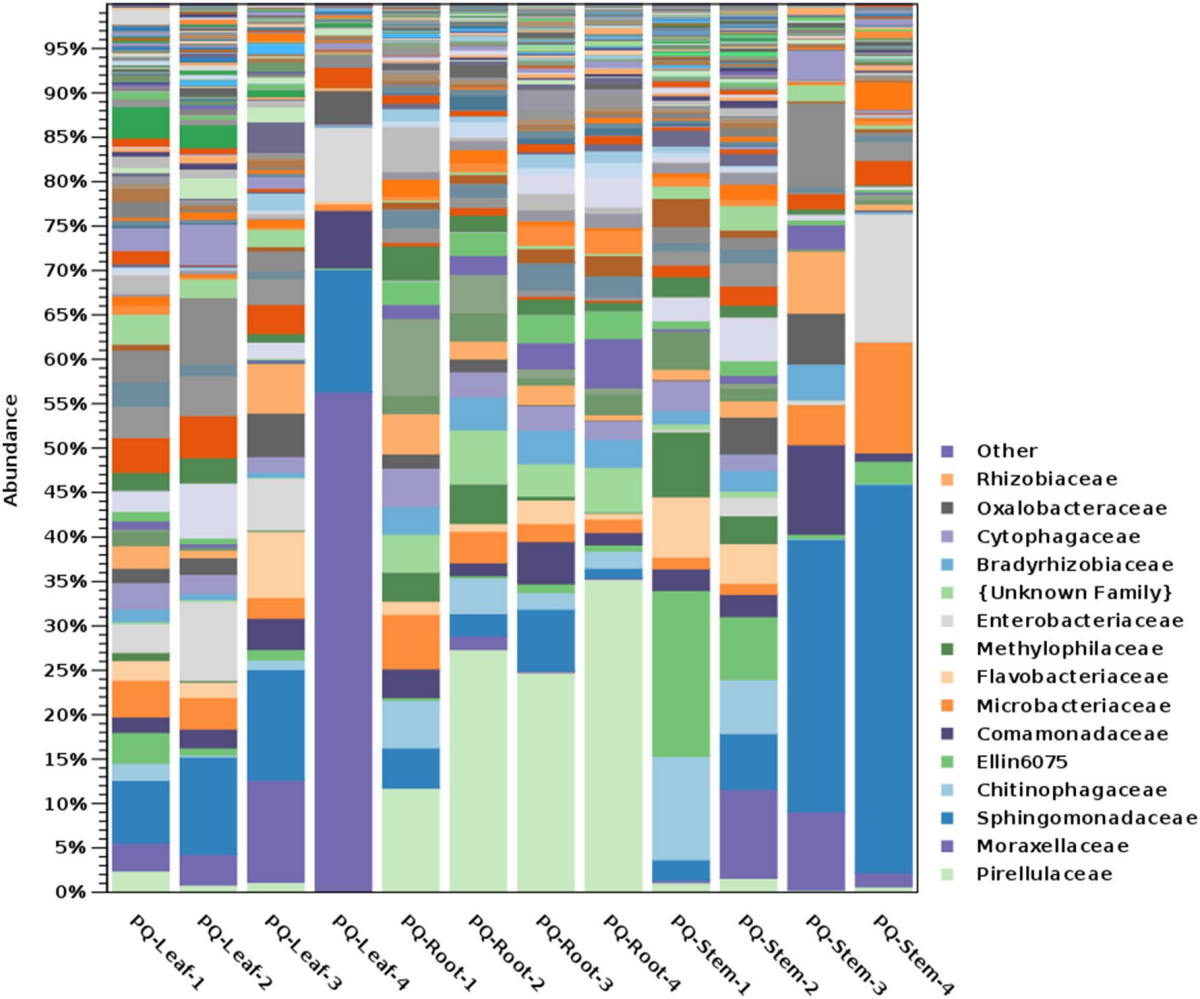
Taxonomic abundance of bacterial families in *Panax quinquefolius* tissues.

At the family level, the most abundant families in GB leaf were Sphingomonadaceae (28%), Cytophagaceae (18%), Oxalobacteraceae (8%), Deinococcaceae (7%) and Methylobacteriaceae (5%) (Fig. 7). The most abundant families in GB stem were Cytophagaceae (25%), Sphingomonadaceae (14%), Sphingobacteriaceae (11%), and Acetobacteriaceae (10%). The most abundant families in GB root were uncultured Acidobacteria family (14%), Bacillaceae (7%), Pirellulaceae (5%), an unidentified Chloroflexi (5%), Nitrososphaeraceae (4%) and Hyphomicrobiaceae (4%) (Fig. 7). On the other hand, the most abundant families in PQ leaf were Moraxellaceae (32%), Sphingomonadaceae (12%), Enterobacteriaceae (7%), and Comamonadaceae (5%). The most abundant families in PQ stem were Sphingomonadaceae (11%), Chitinophagaceae (7%), and Moraxellaceae (6%) (Fig. 8). The most abundant families in PQ root were Pirellulaceae (26%), an unidentified Deltaproteobacteria family (5%) and an unidentified Gammaproteobacteria family (4%) (Fig. 8).

At the level of bacterial genera, the most abundant genera in GB leaf were *Sphingomonas* (27%) and *Hymenobacter* (14%), in GB stem were *Hymenobacter* (16%) and *Sphingomonas* (10%), and in GB root were an unidentified genus in the phylum *Acidobacteria* (14%) and *Bacillus* (5%). The most abundant genera in PQ leaf were *Acinetobacter* (32%), and *Sphingomonas* (11%), in PQ stem were unknown genus in Ellin6075 (9%), *Sphingomonas* (8%), and *Acinetobacter* (6%) and in PQ root were an unidentified Pirellulaceae genus (24%), and an unidentified Myxococcales genus (5%).

### Prediction of functional pathways in GB and PQ associated bacterial communities

**Figure 9:**
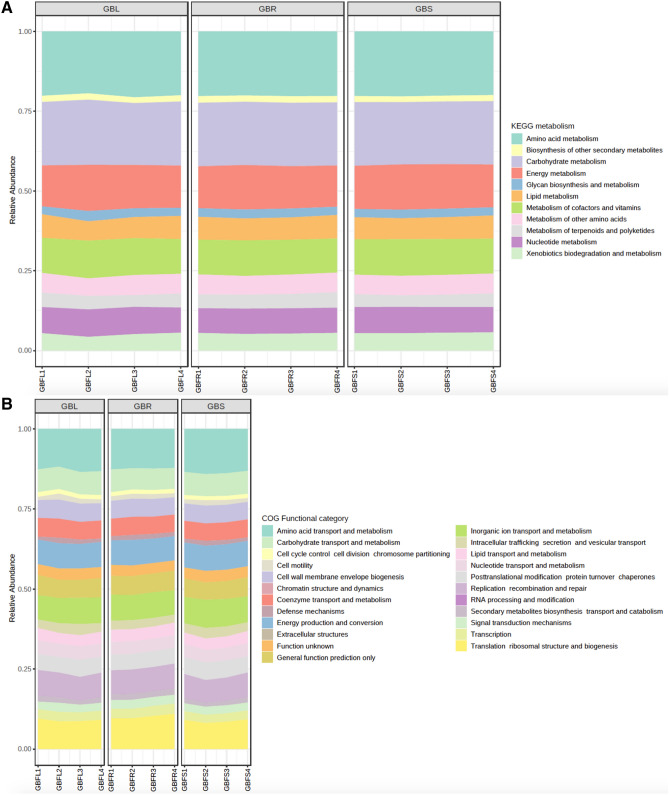
Prediction of functional pathways of *Ginkgo biloba* associated bacterial communities. (**A**) Kyoto Encyclopedia of Genes and Genomes (KEGG) metabolism and (**B**) Clusters of Orthologous Groups of proteins (COG) functional categories.

**Figure 10:**
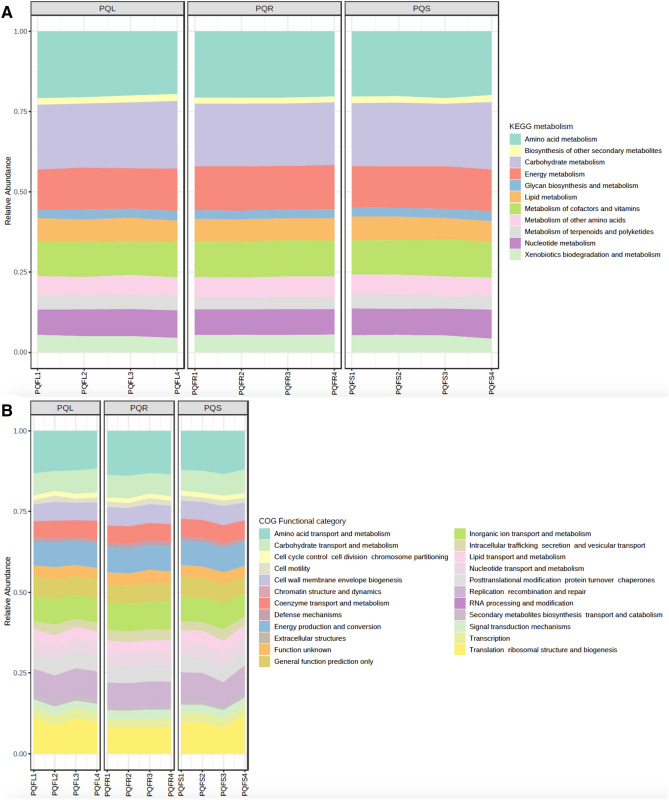
Prediction of functional pathways of *Panax quinquefolius* associated bacterial communities. (**A**) Kyoto Encyclopedia of Genes and Genomes (KEGG) metabolism and (**B**) Clusters of Orthologous Groups of proteins (COG) functional categories.

Diversity analysis using MicrobiomeAnalyst revealed 5843 KEGG orthologs, 140 KEGG pathways, and 11 KEGG metabolism functions in GB, and 6251 KEGG orthologs, 141 KEGG pathways, and 11 KEGG metabolism functions in PQ. The most abundant KEGG metabolism function in GB and PQ were amino acid metabolism, carbohydrate metabolism and energy metabolism (Figs. 9A, 10A). There were 23 predicted Clusters of Orthologous Groups of proteins (COG) in both GB and PQ and the most abundant COG were amino acid transport and metabolism, followed by translation ribosomal structure and biogenesis, inorganic ion transport and metabolism, energy production and conversion, replication, recombination and repair, and carbohydrate transport and metabolism (Figs. 9B, 10B).

Out of 140 KEGG pathways in GB, 71 pathways were predicted at significantly different levels between GB tissues (Supplementary Table S1). Among these pathways are multiple antibiotic biosynthesis pathways, which were significantly more abundant in root compared to stem (tetracycline biosynthesis, penicillin and cephalosporin biosynthesis and vancomycin group biosynthesis). Additionally, multiple xenobiotic biodegradation and metabolism pathways were more abundant in stem compared to root (degradation of chloroalkane and chloroalkene, naphthalene, styrene, atrazine, aromatic compounds, Polycyclic aromatic hydrocarbons).

Out of 141 KEGG pathways in PQ, 80 pathways were predicted at significantly different levels between PQ tissues (Supplementary Table S2). Among these pathways are multiple xenobiotic biodegradation pathways, which were more abundant in root compared to leaf or leaf and stem (chlorocyclohexane and chlorobenzene degradation, fluorobenzoate degradation, polycyclic aromatic hydrocarbon degradation, chloroalkane and chloroalkene degradation, naphthalene degradation, atrazine, and degradation of aromatic compounds). Multiple antibiotic biosynthesis pathways were significantly more abundant in leaf compared to root (penicillin and cephalosporin biosynthesis, macrolides, tetracycline, neomycin, kanamycin and gentamicin biosynthesis).

## Discussion

Plants live in association with communities of microorganisms including fungi, bacteria and Archaea. The relationship between plants and associated microbes can be symbiotic where microbes get access to habitat and nutrients while plants benefit from microbes that can support plant health, growth and disease resistance. Plant associated microbes can be vertically transmitted through seeds from generation to generation, horizontally transmitted between plants or could be acquired from soil^3^. Some plant associated bacteria are tissue specific while others are ubiquitous throughout the plant^3^. In this study, the profiles of the bacterial communities associated with the different tissues of two medicinally and economically important plants (*G. biloba* and *P. quinquefolius*) were characterized using high throughput sequencing*.*

### Alpha diversity of the bacterial communities in the different tissue types

Characterization of the bacterial communities associated with GB and PQ revealed variation in alpha diversity between tissue types (Figs. 1, 2). Higher diversity was observed in GB root compared to leaf and stem. This was not surprising, as the plant rhizosphere is nutrient rich, attracting soil microorganisms to colonize the rhizosphere, resulting in higher colonization in roots^3^. Moreover, roots are protected from harsh environmental conditions such as extreme temperatures, solar radiation and draught, and hence represent a more stable site for associated bacteria^31^. Variation in alpha diversity can also be attributed to the shorter lifespan of leaves that shed every year, compared to established roots where bacterial communities can survive for years or decades^32^. This can also explain the lower bacterial diversity in PQ leaf compared to root and stem according to Simpson’s index and Shannon index.

### Beta diversity of the bacterial communities in the different tissue types

Distinct bacterial communities (Beta diversity) were observed for the different tissues of both GB and PQ (Figs. 3, 4). Bacterial communities clustered by tissue type and composition varied significantly between leaf, stem and root. For PQ, the leaf and stem communities were not well resolved on the first two axes using PCoA but were better resolved using dbRDA (Fig. 4A,B). The Venn diagrams show that some OTUs were ubiquitous throughout the plant while some OTUs were tissue specific (Figs. 3, 4). A higher percentage of ubiquitous OTUs was observed in PQ (49%) compared to GB (13%). The variation in microbiota composition between different plant tissues can be attributed to differing inoculum sources^7^. The leaf and stem associated bacteria can originate from root associated bacteria, but can also be acquired directly from the surrounding environment such as air and rain splash^31^. Other mechanisms underlying this variation are tissue specific environmental selection such as biochemicals, exudates^7^, and tissue specific biotic and abiotic stresses.

### Microbiota profiles in GB and PQ tissues and variability in taxa diversity among the different tissue types

The most abundant phyla in GB and PQ varied by tissue type. The most abundant phylum in both GB leaf and PQ leaf was Proteobacteria followed by Bacteroidetes and Actinobacteria. The major phyla in GB stem were Bacteroidetes, Proteobacteria and Actinobacteria while major phyla in PQ stem were Proteobacteria, Bacteroidetes, Acidobacteria and Actinobacteria. A similar finding was reported from a study of Eucalyptus microbiota where Proteobacteria, Bacteroidetes and Firmicutes were the dominant phyla in Eucalyptus leaf^33^. In another study, Proteobacteria was the most abundant phyla in rice phyllosphere followed by Actinobacteria, Bacteroidetes and Firmicutes^34^. Proteobacteria was also found to be the most abundant phyla in the shoots of the herb *Thlapsi geosingense*^35^. Proteobacteria, Firmicutes, Bacteroidetes and Actinobacteria were the most abundant phyla in romaine lettuce leaf^36^.

The major phyla in GB root were Acidobacteria, Proteobacteria, and Actinobacteria while the major phyla in PQ roots were Proteobacteria, Planctomycetes, Bacteroidetes, and Actinobacteria. In a study of *Arabidopsis thaliana*, Proteobacteria, Bacteroidetes and Actinobacteria were found to be the most dominant phyla in roots^37^. Acidobacteria, the most abundant phylum in GB root, was reported as one of the most abundant bacterial phyla in soil and rhizosphere soil^38^. Acidobacteria were isolated as endophytes from internal plant tissues^35,38^, and were found to have plant growth-promoting effects, possibly mediated by auxin production^38^. Acidobacteria was significantly more abundant in GB root compared to stem and leaf. A similar finding was reported in a study of *Populus* microbiome^7^. This is in agreement with a previous finding that Acidobacteria produce exopolysaccharide during root colonization, which supports its adhesion to root surface^38^. The second most abundant phyla in PQ roots was Planctomycetes. Planctomycetes is a phylum of aquatic bacteria that is present in different habitats such as the oceans, freshwater lakes, wastewater and terrestrial soils^39^. This can explain the significantly higher abundance of this phyla in PQ roots compared to leaf and stem, a finding which was also reported in a study of *Populus* microbiome^7^. Members of Planctomycetes play important role in global carbon and nitrogen cycles^40^.

At the genera level, the genus *Sphingomonas* was abundant in GB leaf and stem and in PQ leaf and stem. In a previous study of *Sphingomonas* in plant leaf, seed and flower tissues*,* the genus was detected at high populations in 26 plant species belonging to 11 families^41^. Other studies reported the isolation of *Sphingomonas* from soybean leaves^42^ and from *Zea mays* stems^43^. *Sphingomonas* is known to be a soil and plant associated bacterium^41^ with nitrogen fixing^44,45^, pathogen resistance^46^, growth promotion^47,48^, as well as xenobiotic degrading abilities^49^.

Another abundant genus in GB leaf and stem is *Hymenobacter.* The genus was previously isolated from the weedy grass *Setaria viridis*^50^, and was identified in stripe rust resistant wheat leaf, suggesting a role in disease resistance^51^. The study suggested direct selection from air by leaves since the genus was reported to be airborne^51,52^. Some species in the genus *Hymenobacter* are radiation resistant, giving them the ability to survive in plant leaves^51,53^. *Hymenobacter* applied to flaxseed and black cumin at seedtime resulted in increased nutritional and functional properties^54^.

The genus *Bacillus* was abundant in GB root. *Bacillus* species were previously isolated from *G. biloba* and were reported to have antimicrobial activities. For example, strains of *Bacillus amyloliquefaciens* isolated from *G. biloba* were found to have antifungal activities against *Phytophthora capsici*^55^. In another study, a *Bacillus subtilis* strain isolated from *G. biloba* was found to have antibacterial activity against foodborne pathogenic bacteria^56^.

### Prediction of functional pathways in GB and PQ associated bacterial communities

The microbiota associated with GB and PQ demonstrated high diversity of functional pathways. This diversity reflects the potential roles these microbes can play as plant symbionts. It was interesting to see higher abundance of antibiotic biosynthesis pathways in bacteria associated with GB root compared to GB stem, and in bacteria associated with PQ leaf compared to PQ root. One of the important mechanisms by which plant associated bacteria promote plant health is combating plant pathogens through the production of antimicrobials. The variation in abundance of antibiotic biosynthesis pathways between plant tissues may suggest a variation in the level of biotic stresses that the different plant niches are exposed to.

A higher abundance of xenobiotic biodegradation pathways was observed in GB stem compared to GB root. A different pattern was observed in PQ where xenobiotic biodegradation pathways were more abundant in root compared to leaf or leaf and stem. Xenobiotic degradation by plant associated bacteria enable bacteria to utilize xenobiotics as a source of carbon and energy while simultaneously helping with environmental bioremediation of these highly persistent contaminants^57^. The variation in abundance of xenobiotic biodegradation pathways between plant tissues may suggest a different level of accumulation of xenobiotic in these plant niches.

Reports on adulteration in NHP are increasing, especially with heavy processing and grinding leading to loss of morphological characteristics^58–60^. Adulteration can be encountered as species substitution, or plant tissue substitution when the therapeutic value is associated with a particular plant tissue (e.g. *Ginkgo biloba* leaves or *Panax quinquefolius* roots). Several chemical and DNA based methods enable detection of species substitution^58,61^, however, there are limited analytical chemistry methods^62,63^ and no DNA tools to differentiate plant tissues. An attractive approach is to investigate the potential of building upon the tissue specificity of some plant associated bacteria that can be used as indicator taxa to identify plant tissues.

## Conclusions

This study characterized the bacterial communities associated with *Ginkgo biloba* and *Panax quinquefolius,* two medicinally and commercially important plant species. The study demonstrated the variation in alpha diversity and beta diversity between bacterial communities associated with different plant tissue types. The most abundant bacterial taxa varied by plant species and by tissue type. *Ginkgo biloba* and *Panax quinquefolius* showed differences in the percentage of ubiquitous versus tissue specific OTUs. Furthermore, the study investigated the functional pathways in bacterial communities associated with *Ginkgo biloba* and *Panax quinquefolius* and highlighted the functional diversity of plant-associated bacteria.

## Methods

### Sample collection and DNA extraction

*Ginkgo biloba* (GB) samples were collected at the Arboretum, University of Guelph, Canada in August 2017, and *P. quinquefolius* (PQ) samples were collected from Victory Ginseng Ontario, Canada in September 2017. Both *G. biloba* and *P. quinquefolius* plants were 2–4 years old, and the habitats where they were sampled were homogeneous habitats. Three tissues were sampled: leaf, stem and root, with four replicates each. Samples were washed before DNA extraction using sterile double distilled water. Root samples were placed in sterile falcon tubes containing sterile double distilled water and tubes were vortexed for 15 s at maximum speed to remove soil particles. Roots were transferred to fresh tubes and washing was repeated until the water remained clear after vortexing^64^. All samples were ground using mortars and pestles before DNA extraction. DNA was extracted from all samples (100 mg from each sample) using Nucleospin^®^ Plant II kit (740770, Macherey Nagel, Germany) according to the manufacturer’s instructions. DNA was quantified using Qubit 3.0 Fluorometer and kept in a − 20°C freezer.

### Amplicon PCR and index PCR

Peptide nucleic acids (PNAs) were previously predicted to be able to block the amplification of host DNA from a range of plants species^65^. To validate the utility of PNAs with *Ginkgo biloba* and *Panax quinquefolius,* the sequences of mitochondrial Peptide Nucleic Acids (mPNA) and plastid Peptide Nucleic Acids (pPNA) were blasted against *G. biloba* and *P. quinquefolius* and they were found to match the sequences corresponding to host organelles at 100% similarity (GB chloroplast: JN867583.1, GB mitochondria: KM672373.1, PQ mitochondria: HM142340.1, PQ chloroplast: KT028714.1). mPNA (MP01-25, PNA Bio) and pPNA (PP01-25, PNA Bio, CA, USA) were used to block the co-amplification of mitochondrial DNA and plastid DNA, respectively.

The 16S V4 region was targeted using 515F and 806R primers, which were modified by adding the Illumina overhang adapter sequences:

515F:TCGTCGGCAGCGTCAGATGTGTATAAGAGACAGGTGCCAGCMGCCGCGGTAA and 806R:GTCTCGTGGGCTCGGAGATGTGTATAAGAGACAGGGACTACHVGGGTWTCTAAT. Primers were purchased from IDT and working solutions were prepared at 5 μM concentrations. Each PCR reaction contained 20 ng of target DNA, 12.5 μl of 2 × KAPA HiFi Hotstart Ready Mix (07958935001, Roche, Canada), 1.25 μl from each of the primers (5 μM), 1.25 μl from each of pPNA and mPNA (10 μM) and up to 25 μl of water. Reaction conditions were 95 °C for 45 s, 25 cycles of 95 °C for 15 s, 78 °C for 10 s, 64 °C for 30 s and 72 °C for 30 s, followed by a hold at 4 °C. Each sample was run in duplicate PCR reactions, which were pooled before the next step. The resulting 16S amplicons were purified using 40 μl AMPure XP beads (A63881, Beckman Coulter, CA, USA) as described in Illumina 16S metagenomics protocol^66^ and were eluted in 25 μl of 10 mM Tris pH 8.5. From each cleaned PCR product, 5 μl were used in index PCR along with 25 μl of 2 × KAPA HiFi Hotstart Ready Mix, 5 μl from each Nextera XT index primer, and 10 μl of PCR grade water. The reaction conditions were 95 °C for 3 min, 8 cycles of 95 °C for 30 s, 55 °C for 30 s and 72 °C for 30 s, then 72 °C for 5 min followed by a hold at 4 °C. The resulting amplicons were purified using 56 μl AMPure XP beads as described in Illumina 16S metagenomics protocol^66^ and were eluted in 20 μl of 10 mM Tris pH 8.5. The expected final PCR product size was ~ 430 bp and was verified using Agilent Bioanalyzer. PCR products were then submitted to The Advanced Analysis Centre (AAC) at University of Guelph for library quantification, normalization and pooling. Libraries were sequenced using MiSeq v3 600 cycle kit, on a 2 × 300 bp run.

### Sequence data analysis

The sequencing data was analyzed using CLC Genomics Workbench (QIAGEN Bioinformatics). The sequence output files were imported, paired, and merged using the following parameters (mismatch cost = 2, minimum score = 8, gap cost = 3 and maximum unaligned end mismatches = 0). Primer sequences were then trimmed from merged reads. Reads were also trimmed based on quality using a quality score limit set to 0.05 and maximum number of allowed ambiguities set to 2. Reads were then trimmed to a fixed length. The resulting reads were used for Operational Taxonomic Units (OTU) assignment. OTU clustering was performed against a reference OTU database (Greengenes V13_5 97%) with similarity percentage set to 97%. OTUs with low abundance (threshold minimum combined abundance of OTU = 50), as well as, OTUs belonging to host DNA were removed. Phylogenetic trees were constructed using Maximum Likelihood approach and then alpha diversity (total number of OTU, Chao-1, Simpson’s index and Shannon’s index) was calculated. Beta diversity (Bray–Curtis) was estimated using MicrobiomeAnalyst^67,68^, and bioVenn tool was used to determine the extent to which OTUs overlapped between the different tissues of GB and PQ (http://www.biovenn.nl)^69^. Bray–Curtis distance-based redundancy analysis (dbRDA), constrained by tissue type, was run using the R package vegan^70^.

### Prediction of functional pathways in GB and PQ associated bacterial communities

MicrobiomeAnalyst was used for functional prediction of the bacterial communities associated with GB and PQ tissues based on 16S rRNA HTS data^67,68^. OTU tables and metadata tables were uploaded to The Marker Data Profiling Module and no extra filtration steps were conducted. Functional prediction was conducted using Tax4Fun function using QIIME against SILVA database Annotation pipeline. The resulting Kyoto Encyclopedia of Genes and Genomes (KEGG) Orthology (KO) table^71^ was imported to the Shotgun Data Profiling Module, and Diversity and Association analyses were investigated.

## Supplementary Information

Supplementary Table S1.

Supplementary Table S2.

## Data Availability

The raw HTS data produced from this study was deposited into the Sequence Read Archive in NCBI under accession number PRJNA726634.
